# 3D Printing Applications in Minimally Invasive Spine Surgery

**DOI:** 10.1155/2018/4760769

**Published:** 2018-04-01

**Authors:** Megan R. Hsu, Meraaj S. Haleem, Wellington Hsu

**Affiliations:** ^1^Department of Orthopedic Surgery, Beaumont Hospital Research Institute, 3811 West 13 Mile Road, Suite #404, Royal Oak, MI 48073, USA; ^2^Department of Orthopedic Surgery, Northwestern University Feinberg School of Medicine, 676 Saint Clair St. Suite #1350, Chicago, IL 60611, USA

## Abstract

3D printing (3DP) technology continues to gain popularity among medical specialties as a useful tool to improve patient care. The field of spine surgery is one discipline that has utilized this; however, information regarding the use of 3DP in minimally invasive spine surgery (MISS) is limited. 3D printing is currently being utilized in spine surgery to create biomodels, hardware templates and guides, and implants. Minimally invasive spine surgeons have begun to adopt 3DP technology, specifically with the use of biomodeling to optimize preoperative planning. Factors limiting widespread adoption of 3DP include increased time, cost, and the limited range of diagnoses in which 3DP has thus far been utilized. 3DP technology has become a valuable tool utilized by spine surgeons, and there are limitless directions in which this technology can be applied to minimally invasive spine surgery.

## 1. Introduction

Additive manufacturing (AM) techniques such as 3D printing (3DP) have been recently used in many disciplines of medicine including the field of spine surgery. More specifically, 3DP pertains to several possible applications in the field of minimally invasive spine surgery including use as biomodels, surgical guides, and implants. 3DP biomodels have the potential to improve preoperative planning and to be used as a valuable teaching tool; surgical guides can increase hardware placement accuracy and precision; implants can be custom designed to fit patient anatomy as well as improve upon the biologic characteristics compared to existing manufacturing methods. This review will delineate the potential for 3DP technology to optimize patient outcomes during minimally invasive spine surgery, as well as current challenges limiting widespread implementation.

## 2. Background

**Figure 1:**
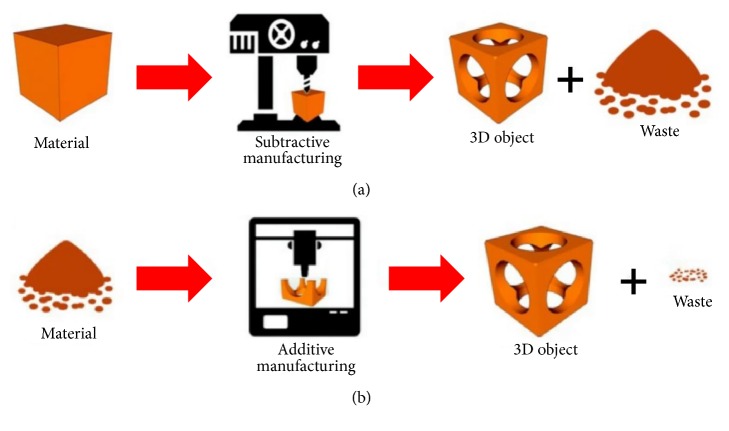
(a) Subtractive manufacturing versus (b) additive manufacturing (source: reproduced and adapted with permission from Ambrosi et al.).

Additive manufacturing such as 3DP utilizes a digital computer-aided design to build a 3-dimensional model by adding successive layers of material rather than through subtractive manufacturing, potentially leading to decreased manufacturing waste (Figure 1) [1].

The idea of using 2D imaging modalities to construct a 3D anatomical model was first described in 1979, and the technology has expanded widely in the medical field since that time [2]. Oral and maxillofacial surgery and orthopedic surgery were two of the first subspecialties to report the use of 3D printing [3]. Its use in the field of spine surgery was first described in 1999 to print models of the entire spine to assist in visualization of complex deformity cases [4].

Since the advent of rapid prototyping (RP), 3DP has become an increasingly valuable adjunct for surgical specialties by facilitating the creation of a wide variety of surgical tools including patient-specific anatomic models, hardware, and cutting guides, as well as implants and prosthetics. As this technology becomes more prevalent, costs are expected to decrease while ease of use will simultaneously increase, and together these factors have the potential to fuel a rapid growth in its adoption [3].

## 3. Biomodels

3DP biomodeling involves the translation of traditional 2D images into a patient-specific anatomic model, which offers several advantages over standard imaging modalities. Izatt et al. quantified orthopedic spinal surgeons' perceptions of the usefulness of biomodels compared to standard 2D imaging modalities in treating patients with either complex spinal deformities or spinal tumors. The biomodels were used both for preoperative planning and intraoperative anatomical reference, and it was reported that anatomical details were more visible on the biomodel in 65% of cases and exclusively visible on the biomodel in 11% of cases [5]. Furthermore, use of a 3D biomodel preoperatively led to alternative decision-making regarding the choice of materials used in over half of the cases and the implantation site in 74% of cases and reduced operating time by an average of 22% [5]. These data support biomodeling as a useful, and sometimes essential, imaging tool used for complex spinal surgery.

**Figure 2:**
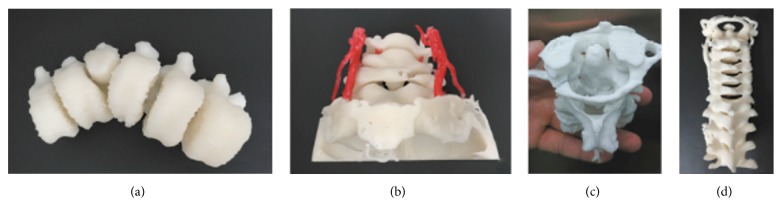
(a) Scoliosis. (b) Atlas neoplasm. (c) and (d) Cervical fracture-dislocation (source: reproduced and adapted with permission from Wang et al.).

Biomodeling in spine surgery has the potential to play a significant role in preoperative planning. The ability to interact with a patient's anatomy in a tactile manner prior to the procedure itself produces several tangible benefits including reduced operative time, lower blood loss, and reduced transfusion volumes [6–8]. Furthermore, the creation of such biomodels allows surgeons to optimize their intraoperative hardware placement prior to the time of surgery, especially in patients with complex anatomical pathologies including rheumatoid arthritis and complex scoliosis [6, 9, 10]. A retrospective review compared 50 patients who had 3DP spinal biomodels composed of polystyrene created prior to surgical correction of Lenke Type 1 adolescent idiopathic scoliosis with 76 patients who received the standard of care (no biomodeling) and showed that the treatment group had a significantly (*p* = 0.02) decreased rate of pedicle screw misplacement in patients with a Cobb angle of >50 degrees [6]. Additionally, 4 complex patients who had congenital scoliosis, an atlas neoplasm, atlantoaxial dislocation, and an atlantoaxial fracture-dislocation were treated with the aid of 3D printed photosensitive resin biomodels created prior to surgery (Figure 2) [11].

Postoperative imaging in these 4 patients demonstrated that no pedicle penetration or screw misplacement took place, again demonstrating the use of biomodels to assist surgeons in treating patients suffering from complex anatomical pathologies.

**Figure 3:**
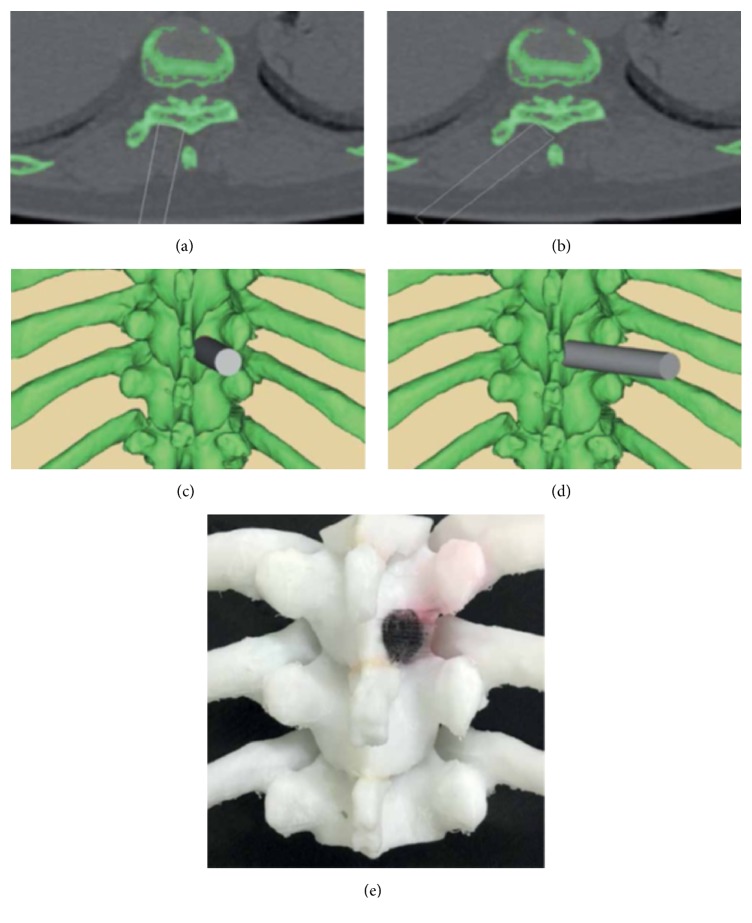
Surgical simulation demonstrating virtual channel placement (a–d). 3DP thoracic vertebra for preoperative planning (e) (source: reproduced and adapted with permission from Zhao et al.).

Minimally invasive spine surgery (MISS) has unique clinical challenges surgeons encounter on a daily basis, such as small exposure corridors, difficult visualization, minute working spaces, and a steep learning curve paired with low tolerability for error [12]. In response, biomodels can provide MIS surgeons with tactile feedback and facilitate the means to understand complex patient anatomy during the preoperative planning phase. One example of this application is treatment of thoracic ossification of the ligamentum flavum (TOLF) using biomodel assisted MISS. This approach was utilized in a study of 13 patients who each had 3D biomodels of their spinal anatomy created prior to microsurgical decompression of TOLF (Figure 3) [13]. The biomodel was utilized to determine anatomical variations between patients, preoperatively optimize the angle of insertion of percutaneous tubular retractors, and delineate the location and size of the relevant bony spaces to reduce damage to adjacent muscles, tendons, and bones.

A similar approach was taken to assist in the case of a 66-year-old man with T10-T12 OLF in whom a biomodel was printed during the preoperative planning phase. The surgeons utilized this model to verify their osteotomy angle, as well as confirm the size, location, and boundaries of the OLF [14].

Because the successful mastery of MISS skills requires a thorough understanding of 3D spinal architecture, there is a steep learning curve for these procedures [12]. Potential complications include durotomy, implant malpositioning, and neural injury. 3DP biomodels have the potential to play an important role in the training of new surgeons by illuminating the intricate anatomy and architecture of the spine that cannot be simulated using alternate modalities. By providing real-time, tactile feedback, these models can accelerate the comfort and familiarity of early adopting surgeons in working within this space. For example, published experiences in the use of 3DP models in other diagnoses such as aortic aneurysms have led to better scores on a preoperative assessment compared to counterparts who used traditional CT imaging [15].

## 4. Guides and Templates

**Figure 4:**
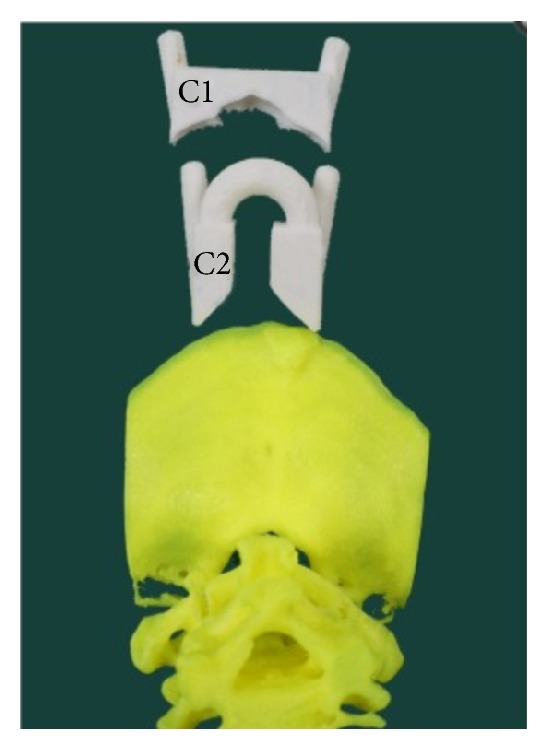
3DP drill guide custom designed to fit a biomodel of 57-year-old male with atlantoaxial dislocation (source: reproduced and adapted with permission from Guo et al.).

**Figure 5:**
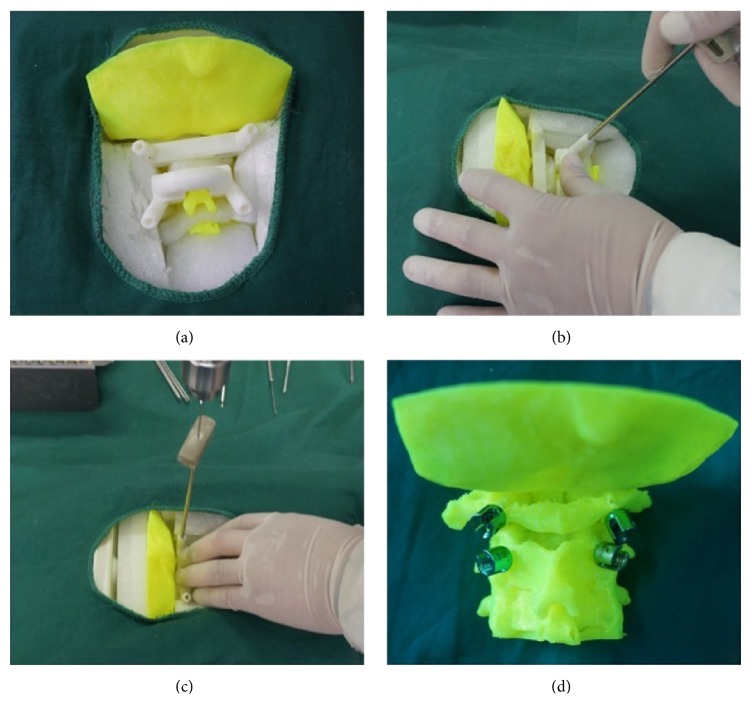
Navigation template-assisted pedicle screw fixation in upper cervical spine. (a) Navigation template pressed to fit with vertebra. (b) Placement of navigation protective sleeve. (c) Kirschner wire perforated along protective sleeve. (d) Pedicle screw fixation (source: reproduced and adapted with permission from Guo et al.).

3DP can facilitate the creation of patient-specific guides and templates, which can aid preoperative planning thereby increasing the accuracy and precision of hardware placement intraoperatively. The mechanism for this usually utilizes a computed tomography scan of the spine that can then be translated into a 3DP guide or template. Potential benefits include shorter operating room times and reduction of radiation exposure to the patient and the surgical team [8, 16–18]. Also, 3D-printed screw placement guides have demonstrated superior accuracy in the placement of pedicle and laminar screws in several studies, thereby increasing patient safety and clinical outcomes [17, 19, 20]. In one study by Merc et al., the incidence of cortex perforation was found to be significantly reduced in the group utilizing a 3DP template versus free-hand screw placement under fluoroscopic guidance [21]. Furthermore, a study by Lu et al. utilized a reverse engineered biomodel and 3DP lumbar pedicle drill guide, which was used to place screws in 6 patients [22]. The drill template precisely fit over all patients' anatomy, allowing for rapid template positioning, drilling, and screw placement. Postoperative CT imaging demonstrated a high degree of precision and accuracy as all drill trajectories and screw placements were found to be in their optimal locations. Another study by Guo et al. compared the efficacy of using 3DP guided screw placement in the upper cervical spine (atlas and axis) versus traditional placement under fluoroscopy (Figures 4 and 5) [23].

Screws placed using the 3DP guide demonstrated increased placement accuracy with reduced pedicle cortex perforation and reduced operation time and fluoroscopic frequency when compared with traditional placement. Together, these studies demonstrate the benefits of 3DP templates, which include efficient, accurate, and precise drill trajectories and screw placement, which can lead to faster surgery times and improved patient outcomes.

3DP templates and guides have a number of applications to minimally invasive spine procedures. Specific examples of such applications include minimizing exposure sites and incision size, navigating variable patient anatomy, and patient-specific instrumentation. First, 3DP may allow for smaller templates and guides, which in conjunction with optimized drill trajectories would promote smaller incisions and exposures. The ultimate goal in this arena would be the construction of a device that fits externally on the patient's body that guides drill trajectories and screw placement without the need for invasive exposures. However, drawbacks do exist with the use of smaller, more precise instruments. For example, although smaller guides and templates minimize native tissue disruption, there is the simultaneous risk that smaller exposures and instrumentation may preclude proper hardware fit and placement. Surgeons utilizing minimally invasive techniques should be aware of the tradeoffs that exist in using these tools.

Another critical application to MISS is within the arena of craniocervical surgery, where anatomical structures can be highly variable between patients [24]. Drill templates and trajectories that can be optimized prior to surgery would be invaluable in navigating within this intricate anatomical space and could mitigate the risks of vertebral artery injury, a potentially life threatening complication. A future application of 3DP guides and templates may be patient-specific instrumentation to work specifically within the unique architecture of each patient's anatomy. One possible example would be the introduction of biologics in a small posterior interbody space, where a surgeon could use 3DP instruments that are unique to the size of that patient's disc and opening space. The specificity of such instruments may facilitate the use of MISS in cases where patient anatomy precluded the use of such techniques beforehand. In all of these examples, the use of 3DP can ease the difficulty of placing hardware or biologics in compressed spaces and under limited visibility.

## 5. Implants

3D printing can also contribute to MISS by creating custom-designed and patient-specific implants for insertion. Furthermore, these processes allow for fine tuning of material characteristics and can serve as a platform for tissue engineered scaffolds to promote bone healing [25, 26].

**Figure 6:**
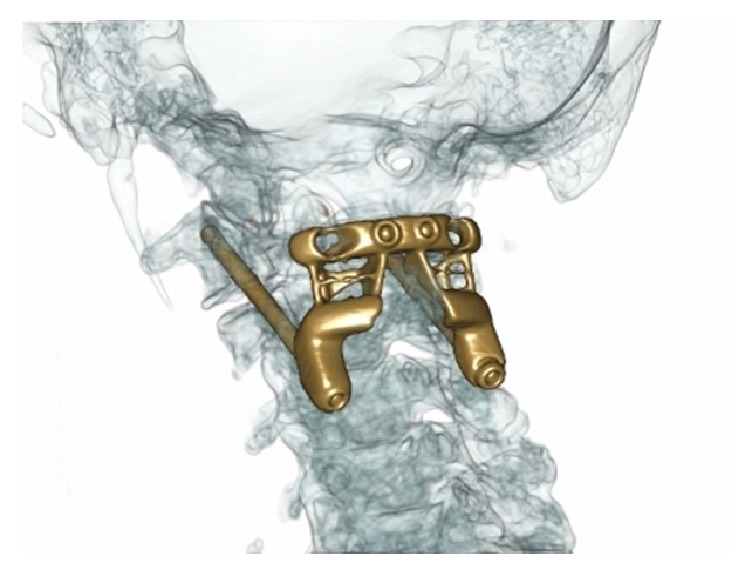
CT with proposed 3DP titanium C1-C2 prosthesis overlaid (source: reproduced and adapted with permission from Phan et al.).

**Figure 7:**
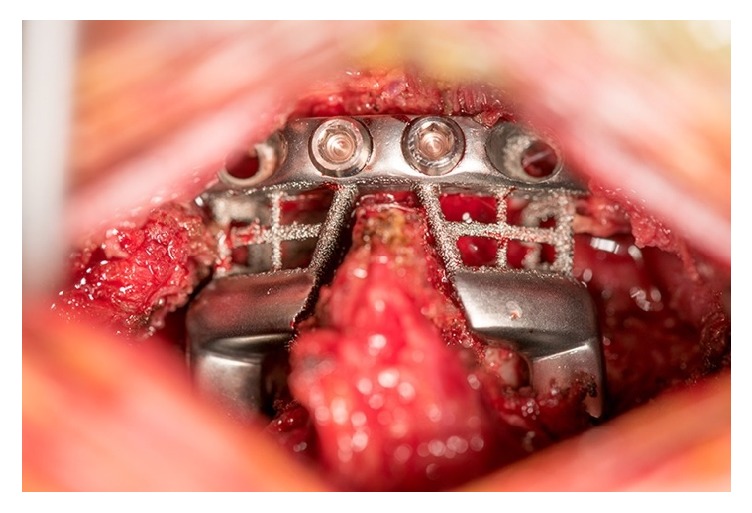
Intraoperative implantation of 3DP custom printed spinal prosthesis for a posterior C1-C2 fusion (source: reproduced and adapted with permission from Phan et al.).

Examples include a custom printed spinal prosthesis for a posterior C1-C2 fusion in a 65-year-old female patient as well as a prosthesis to reconstruct the C2 vertebrae in a pediatric patient with ewing sarcoma (Figures 6 and 7) [27, 28].

In another case, investigators created a custom spinal fusion cage with patient-specific dimensions using the patient's CT scan data [29]. The goal was to create an implant that was custom fit to the patient's vertebral body endplates. Researchers first made a 3D reconstruction of the patient's spine from their CT DICOM and subsequently restored sagittal balance by adjusting the lordotic angle of the proposed implant. The 3D model also allowed for simulated osteophyte removal and implant placement preoperatively. The custom-designed titanium cage was manufactured using additive manufacturing direct metal printing (DMP) technology. In all cases, patient-specific implant rendering can maximize anatomic fit of the device and minimize the chance of implant drift or subsidence.

3DP implants can also be created from different materials with customizable stiffness and porosity, allowing them to maximize bony ingrowth and osseointegration [30]. McGilvray et al. directly compared the bony ingrowth potential and biomechanical properties of a novel 3DP porous titanium alloy (PTA) interbody cage with commercially available polyetheretherketone (PEEK) and plasma sprayed porous titanium coated PEEK (PSP) interbody cages in an ovine lumbar interbody fusion model [31]. Investigators reported a statistically significant decrease in flexion-extension range of motion upon biomechanical testing, and a statistically significant increase in stiffness in the PTA cages compared to PEEK and PSP cages (*p* = 0.02 and *p* ≤ 0.01, resp.). MicroCT revealed a statistically significant increase in bone volume (*p* < 0.01) of PTA cages compared to PEEK and PSP cages at 8-week and 16-week time points. Authors attribute these findings to increased peri-implant osteogenesis evident on histomorphometric analysis, theorizing that increased ingrowth across the osteoconductive surface provided by the 3DP PTA cage contributes to increased implant stability and fusion-promotion.

Investigative 3DP spinal implants have been applied to fusion, artificial disc, and SI joint implants. In a proof of concept model with the goal of reducing reoperation rates in minimally invasive SI joint fusion, investigators compared a 3D-printed, additive manufactured (AM), porous triangular implant with a solid titanium plasma spray (TPS) porous coated implant in a bilateral ovine distal femoral defect model. They found that AM implants displayed significantly more bony ingrowth into the device's core versus superficial ingrowth with the TPS coated implant. Authors suggest the increased porosity of AM implants more closely mimics native bone and may enhance the biomechanical stability in MIS SI joint fusion [32].

Finally, this manufacturing technology has been used to promote bone regeneration without the need for autografts, allografts, or exogenous factors such as BMP-2. The use of autografts and allografts is limited due to donor site pain, increased morbidity, limited availability, and the potential for disease transmission, while rh-BMP2 can cause significant complications at supraphysiologic doses [33–35]. Jakus et al. utilized 3DP to produce hyperelastic “bone” (HB) which is a synthetic biomimetic with similar elastic properties to native bone. In this study, HB not only supported cell growth, but also promoted differentiation of BMSCs* in vitro* without the aid of osteogenic factors in the culture media. Jakus et al. additionally found higher mean fusion scores with a HB scaffold in a rat PL fusion model versus a collagen scaffold control [36]. The ability to 3D-print a synthetic, growth-factor-free material may represent a superior and safer method to regenerate bone than existing techniques.

These examples provide opportunity for the MIS surgeon. Given the constricted working corridors, the ability to create hardware and biologics specific to the patient's anatomy could prove invaluable. The capacity to fine-tune the osteoinductive nature of implantable materials would decrease pseudarthrosis rates, which is a known complication from these types of procedures. The reduction of operative times, the need to retract surrounding neural structures, and nonunion rates are all exciting potential benefits of this technology for the MIS surgeon.

### 5.1. “Off-the-Shelf” Implants

In addition to its uses to develop custom, patient-specific implants, 3DP technology is also being used to optimize the geometric properties of premade “Off-the-Shelf” (OTS) implants [37]. As described previously by McGilvray et al., the customizable porosity and stiffness of 3DP materials can more closely mimic native bone and facilitate bony ingrowth [30–32]. OTS products are created by premanufacturing implants with a wide variety of sizes and fits, thus reducing the additional time required for additional imaging studies and custom implant printing [37]. Therefore, the core appeal of 3DP implants, namely, their customizable porosity and dimensions, is maintained, while also making 3DP technology more cost-effective and less time intensive. In all, this process would make 3DP implants more widely available, while still conferring the unique structural advantages offered by this emerging technology.

## 6. Limitations

Despite the benefits conferred by the use of 3DP in surgery, barriers remain that have precluded its widespread adoption. Among the most significant of these barriers appears to be the additional cost incurred in utilizing 3DP technology (including start-up, cleaning, and maintenance), the time required to develop the 3DP device, and the lack of data supporting the use of 3DP for routine procedures [38, 39]. 3DP remains a highly specialized process that requires significant capital investments in complex design software, cameras, and the 3D printing machine itself [39]. The price of such an investment may reduce a hospital's willingness to adopt 3DP as well as inflicting a prohibitive cost burden upon the patient. By reducing the demand for such technology from both the hospital and patient side, the financial burdens of 3DP have severely curtailed its adoption on a wider scale.

Additionally, the amount of time required to develop 3DP devices is not insignificant. The process for creating a single device for a patient may involve additional imaging procedures, development in the 3D modeling space, and the printing of the device itself [39]. These lengthy time requirements may deter patients who cannot or will not tolerate additional time spent in a clinical environment.

Lastly, the benefits for 3DP in spine surgery have thus far been limited to complex cases that are handled by a very limited number of specialized surgeons [38]. This technology has provided surgeons with an unparalleled ability to provide patient-specific interventions in such cases. However, not all spine surgery cases possess the complexity to require this degree of specificity, and the benefits provided by 3DP are not always translatable. As such, 3D printing technology currently exists in relatively niche cases, which reduces its market potential and generalizability and limits its overall use.

## 7. Conclusion

The use of 3D printing in the field of spine surgery is rapidly evolving, including its emerging use to enhance the field of minimally invasive spine surgery. Potential applications are myriad and include biomodels, surgical guides, and implants. Biomodels can assist with preoperative planning, mitigate the complications incurred during the initial learning curve associated with MISS, and serve to increase patient understanding and satisfaction. 3D printed surgical guides can improve accuracy and specificity in hardware placement. Lastly, 3D-printed implants promote superior fit and osteoinductivity, which work synergistically with the principles of minimal disruption that underlies MISS. Although cost, time, and the relatively specialized market are currently inhibiting widespread adoption of 3DP technology, it is nonetheless a valuable area that merits ongoing research.
